# Sequence Factorization with Multiple References

**DOI:** 10.1371/journal.pone.0139000

**Published:** 2015-09-30

**Authors:** Sebastian Wandelt, Ulf Leser

**Affiliations:** Knowledge Management in Bioinformatics, Humboldt-University of Berlin, Rudower Chaussee 25, 12489 Berlin, Germany; Bangladesh University of Engineering and Technology, BANGLADESH

## Abstract

The success of high-throughput sequencing has lead to an increasing number of projects which sequence large populations of a species. Storage and analysis of sequence data is a key challenge in these projects, because of the sheer size of the datasets. Compression is one simple technology to deal with this challenge. Referential factorization and compression schemes, which store only the differences between input sequence and a reference sequence, gained lots of interest in this field. Highly-similar sequences, e.g., Human genomes, can be compressed with a compression ratio of 1,000:1 and more, up to two orders of magnitude better than with standard compression techniques. Recently, it was shown that the compression against multiple references from the same species can boost the compression ratio up to 4,000:1. However, a detailed analysis of using multiple references is lacking, e.g., for main memory consumption and optimality. In this paper, we describe one key technique for the referential compression against multiple references: The factorization of sequences. Based on the notion of an optimal factorization, we propose optimization heuristics and identify parameter settings which greatly influence 1) the size of the factorization, 2) the time for factorization, and 3) the required amount of main memory. We evaluate a total of 30 setups with a varying number of references on data from three different species. Our results show a wide range of factorization sizes (optimal to an overhead of up to 300%), factorization speed (0.01 MB/s to more than 600 MB/s), and main memory usage (few dozen MB to dozens of GB). Based on our evaluation, we identify the best configurations for common use cases. Our evaluation shows that multi-reference factorization is much better than single-reference factorization.

## 1 Introduction

The development of novel high-throughput DNA sequencing techniques has led to an exponentially increasing flood of data. Only one/few individuals of each species was sequenced (like humans, mice, E.coli, etc.) until recently. Decreasing costs now make it possible to sequence large samples of a given population. Examples for such projects are the 1000-Genomes project [1]; the international cancer sequencing consortium [2]; the UK10K project [3], and the Million Veteran Program [4]. These large-scale projects are generating comprehensive surveys of the genomic landscape of phenotypes (or diseases) by sequencing thousands of genomes [5]. Managing, storing and analyzing this quickly growing amount of data is challenging [6]. Sequence compression is a key technology to cope with the increasing flood of DNA sequences [7].

Compressing DNA with standard compression techniques is ineffective, since the distribution of symbols is close to random. Accordingly, standard compression schemes can often reduce the space requirements by only approx. 6:1 (one base is encoded with up to 1.3 Bits), see [8–10] for recent surveys. However, if a project only considers genomes from one species, scientists often deal with hundreds of highly similar genomes. Similarity between biological sequences can be exploited for compression using so-called referential compression schemes [11–16], which encode the differences between an input sequence and a reference sequence. Since the reference sequence is assumed to be transferred separately, the overall size of compressed deviations (encoded pointers into the reference) is smaller than the uncompressed size of a sequence. Such referential compression techniques with single references achieve compression ratios of 1,000:1 and more for Human genomes. Preliminary experiments showed that referential compression against multiple references often further boosts compression ratios [17], for instance up to 4,000:1 and higher for human genomes [18, 19]. Intuitively, the more references are present, the longer matches can be found in the collection, and the fewer compressed entries (=factors) are required for storage of sequences. However, detailed analysis of multi-reference referential factorization is an open problem. Open questions include: How much gain does one obtain when computing a factorization against multiple references? How close to optimal are approximate factorization algorithms, which need considerably less main memory? Which technique/index should be applied to compute a multi-reference factorization under resource constraints (bandwidth limits and required main memory)? With this paper we provide an in-depth analysis of multi-reference factorization techniques. Our major contributions are as follows:

1. We define the notion of an **optimal factorization** against multiple references, based on the number of pointers into reference sequences. We use this measure to evaluate a multitude of different factorization algorithms.
2. We devise three basic algorithms for computing an optimal factorization, each with different runtime characteristics. While the first algorithm is history-free, i.e. it factorizes all sequences independently from each other, the other two algorithms **keep track of previously factorized sequences** and **exploit** this information to speed up optimal factorization.
3. We develop a reference extension technique, which **factorizes against multiple references, while only an index over a single reference is needed**, and thus requiring significantly less main memory.
4. We perform an exhaustive evaluation on **30 configurations**, which are instantiations of our general factorization framework, on **six datasets from three species** (Human, Arabidopsis thaliana, and Yeast).
5. Our results show a wide range of factorization rates (optimal to an overhead of up to 300%), factorization speed (0.01 MB/s to more than 600 MB/s), and main memory usage (few dozen MB to a dozens of GB). We identify the **best configurations depending on common use cases** using multi-criteria decision analysis techniques.

### Multi-reference referential factorization

We first revisit some preliminaries for referential compression and factorization as in [18]: A *sequence* *s* is a finite ordered list of characters from an alphabet Σ. The concatenation of two sequences *s* and *t* is denoted with *s* ∘ *t*. A sequence *s* is a *subsequence* of sequence *t*, if there exist two sequences *u* and *v* (possibly of length 0), such that *t* = *u* ∘ *s* ∘ *v*. The length of a sequence *s* is denoted with ∣*s*∣ and the subsequence starting at position *i* with length *n* is denoted with *s*(*i*, *n*). *s*(*i*) is an abbreviation for *s*(*i*,1). All positions in a sequence are zero-based, i.e., the first character is accessed by *s*(0). A referential factorization of a sequence encodes the sequence as a concatenation of subsequence-pointers into references. We extend the definition of referential factorization from a single reference [18] to multiple references:

**Definition 1 (Referential Factorization)** *Let REF be a set of reference sequences and* *s* *be a to-be-factorized sequence. A quadruple* *rme* = (*id*, *s*, *l*, *m*) *is called* *referential match entry* (RME) *if* *id* ∈ *REF* *is a (identifier for a) reference sequence*, *s* *is a number indicating the start of a match within* *id*, *l* *denotes the match length, and* *m* *denotes a mismatch symbol. A* *referential factorization* *of* *s* *w.r.t*. *REF* *is a list of RMEs* *f* = [(*id*_1_, *s*_1_, *l*_1_, *m*_1_),…, (*id*_*n*_, *s*_*n*_, *l*_*n*_, *m*_*n*_)] *such that* (*id*_1_(*s*_1_, *l*_1_) ∘ *m*_1_) ∘ … ∘ (*id*_*n*_(*s*_*n*_, *l*_*n*_) ∘ *m*_*n*_) = *s*.

The *size* of a RME (*id*, *s*, *l*, *m*) is defined as *l*+1. The *offset* of a RME *rme*_*i*_ in a referential factorization *f* = [*rme*_1_,…, *rme*_*n*_], denoted *offset*(*f*, *rme*_*i*_), is defined as ∑_*j* < *i*_∣*rme*_*j*_∣.

The algorithm shown in Table 1 is a simple method for computing a referential factorization against multiple references. The input sequence *s* is traversed from left to right, and at each step, the longest prefix of *s* which can be found in any of the references {*ref*_1_,…, *ref*_*m*_}, is replaced with one RME. Unfolding a factorized sequence *f* is equally simple: We replace each RME in *f* with its unfolded sequence, where the unfolding of a single RME *rme* = (*id*, *s*, *l*, *m*) is *id*(*s*, *l*) with the mismatch character *m* concatenated to the end. The factorization of *s* against *REF* is denoted with *f* = *fact*(*s*, *REF*) and the unfolding of *f* is denoted with *s* = *unfold*(*f*, *REF*).

**Table 1:** Optimal algorithm for computing a referential factorization against multiple references.

	**Input:** to-be-factorized sequence *s* and collection of reference sequences *REF* = {*ref*_1_,…, *ref*_*m*_}
	**Output:** factorization *f* of *s* with respect to *REF*
1:	Let *f* be an empty list
2:	**while** ∣*s*∣ ≠ 0 **do**
3:	Let *pre* be the longest prefix of *s*, such that $(pos,pre)\in search{\left(ref_{i}\right)}_{pre}^{0}$, for a number *pos*, and there exists no 1 ≤ *j* ≤ *m*, with *j* ≠ *i* and *ref*_*j*_ contains a longer prefix of *s* than *ref*_*i*_
4:	**if** *s* ≠ *pre* **then**
5:	Add (*ref*_*i*_, *pos*,∣*pre*∣, *s*(∣*pre*∣)) to the end of *f*
6:	Remove the first ∣*pre*∣+1 symbols from *s*
7:	**else**
8:	Add (*ref*_*i*_, *pos*,∣*pre*∣ − 1, *s*(∣*pre*∣ − 1)) to the end of *f*
9:	Remove the prefix *pre* from *s*
10:	**end if**
11:	**end while**

It should be noted that computing a factorization against a set of references can also be achieved by first concatenating all reference sequences in *REF* to a single sequence *ref* and then compute a factorization against *ref*. In this case, the factorization can be computed using standard factorization algorithms, e.g. [20–24]. Implementations of compression algorithms against a single reference, e.g. GDC [13] and RLZ [25], can be applied to multiple references in a similar way. The major limitation of these algorithms/implementations is that similarities between references are left unexploited. We would like to further emphasize that an optimal factorization does not always result in an optimal compression, since the factorization is compressed by an entropy encoder or using heuristic-based encodings. However, a small factorization is usually a prerequisite for achieving high compression rates.

**Fig 1:**
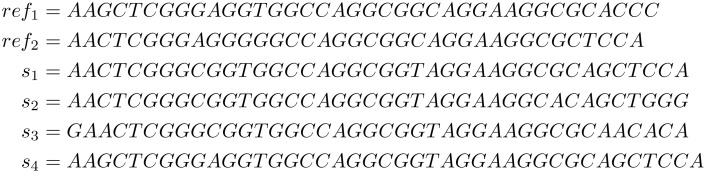
Sequences for Example 1.

**Example 1** *Given the sequences in Fig 1, let* *REF* = {*ref*_1_, *ref*_2_}. *We obtain the following referential factorizations with the algorithm from Table 1*:

$$\begin{array}{cc} fact(s_{1},REF)= & \left[(1,0,8,C),(0,10,13,T),(0,24,11,71),(1,32,4,A)\right]\\ fact(s_{2},REF)= & \left[(1,0,8,C),(0,10,13,T),(0,24,8,A),(0,23,3,67),(0,12,3,G)\right]\\ fact(s_{3},REF)= & \left[(0,26,3,C),(0,4,5,C),(0,10,13,T),(0,24,11,65),(0,33,3,A)\right]\\ fact(s_{4},REF)= & \left[(0,0,23,T),(0,24,11,G),(1,32,4,65)\right] \end{array}$$

We define a factorized sequence database for a collection of sequences as follows.

**Definition 2 (Factorized sequence database)** *A* factorized sequence database (FSD) *for a collection of sequences* *S* = {*s*_1_,…, *s*_*n*_} *and a collection of references* *REF* = {*ref*_1_,…, *ref*_*m*_}, *is a collection of referential factorizations* *fsd* = {*f*_1_,…, *f*_*n*_}, *such that for all* 1 ≤ *i* ≤ *n*:*unfold*(*f*_*i*_, *REF*) = *s*_*i*_. *The size of a factorized sequence database is* *size*(*fsd*) = ∑_*f* ∈ *fsd*_∣*f*∣. *A factorized sequence database* *fsd is* minimal *for a collection of sequences S and a collection of references REF*, *if there exists no fsd*_2_ *for S and REF with size*(*fsd*_2_) < *size*(*fsd*). *The* FSD size optimization problem *is to find a minimal fsd for S and REF*.

Note that the factorized sequence database does not take into account the size of the references, but only the size of factorizations. Considering the size of the references in addition, is an interesting direction for future work. In the following, we first prove that the algorithm in Table 1 computes minimal factorized sequence databases (Proposition 1). We analyze the complexity of this algorithm in Proposition 2.

**Proposition 1** *Given *S* = {*s*_1_,…, *s*_*n*_} and *REF*, let *f*_*i*_ be the result of algorithm in Table 1 for sequence *s*_*i*_ and references *REF*. The factorized sequence database {*f*_1_,…, *f*_*n*_} is minimal as long as the storage necessary for a single RME is uniform*.

**Proof 1** *Since we use suffix dictionaries and traverse the input sequences from left-to-right, the same arguments from Proof 1 in [26] are used to prove optimality. Note that a prerequisite for optimality is that the size of a referential match entry is constant*.

**Proposition 2** *Given *S* = {*s*_1_,…, *s*_*n*_} and *REF*, a minimal factorized sequence database for *S* and *REF* can be computed in* *O*(n*∣*REF*∣*max_*s*_*i*_ ∈ *S*_(∣*s*_*i*_∣)).

**Proof 2** *Each of the *n* sequences from* {*s*_1_,…, *s*_*n*_} *is factorized separately. The algorithm from Table 1 factorizes a single sequence in* *O*(∣*REF*∣*max_*s*_*i*_ ∈ *S*_(∣*s*_*i*_∣)) *as follows: The longest matching prefix *pre* of the to-be-factorized sequence *s*_*i*_ is looked up in each reference* (∣*REF*∣ *in total*). *This takes* *O*(∣*pre*∣*∣*REF*∣) *time, using an index structure for each reference which allows subsequence lookups in time linear to the query length. After the longest prefix is identified, at least ∣*pre*∣ symbols are removed from the beginning of *s*_*i*_ and the process is repeated. Thus, we have* *O*(∣*REF*∣*∣*s*_*i*_∣) *lookups for sequence *s*_*i*_. Overall, the number of lookups for a single sequence in *S* is bound by* *O*(∣*REF*∣*max_*s*_*i*_ ∈ *S*_(∣*s*_*i*_∣)). *Since we have *n* sequences, we obtain* *O*(n*∣*REF*∣*max_*s*_*i*_ ∈ *S*_(∣*s*_*i*_∣)).

Note that Proposition 2 can equally be stated and proven over the concatenation of all references into one sequence, e.g. if we create a single suffix tree over the concatenation of all references. However, in practice, one suffix tree per reference is more convenient. First, many implementations have difficulties to handle input sequences with several billion characters, since during index construction these techniques need main/external memory 10–15 times the length of the concatenated sequences. Second, whenever a reference is changed/removed/added, the suffix tree over the whole concatenation needs to be recomputed.

## 2 Methods

The computation of a minimal factorized sequence database is time consuming for very long sequences. The actual run time depends heavily on the number of lookups in the index structures of the references. In Section 2.1, we discuss how to increase the efficiency of factorization techniques in terms of speed, by exploiting information about previously compressed sequences to reduce the number of index lookups. In Section 2.2, we discuss how to avoid keeping all reference indexes in main memory. We design a technique called reference extension, which rewrites a single-reference referential factorization into a referential factorization against multiple references, based on a compressed representation of references.

### 2.1 Avoiding index lookups

The factorization speed against multiple references mainly depends on the number of index lookups during factorization: While the overall factorization process is linear in the length of the sequence, the lookup of a to-be-factorized prefix in the references is very time consuming. For instance, walking down a suffix tree character by character will frequently yield a cache miss, and can easily take up to several milliseconds for long references, if long prefixes match. In contrast, performing character-wise sequence matching from fixed positions in two sequences is very fast on today’s computer architectures. In the algorithm from Table 1, which computes an optimal factorization, we need to perform a lookup in the reference indexes for each new RME. Finding the longest matching prefix is necessary to guarantee optimality.

We design three heuristics, which avoid index lookups by exploiting the factorization context, i.e., the history of the factorization so far. In order to discuss our heuristics, we define a general algorithm for factorization of a sequence database against a collection of references. The algorithm is shown in Table 2. This algorithm is an extension of the algorithm from Table 1, with the following differences. First, we factorize a whole sequence database, not only a single sequence. Second, we keep track of the previously encoded RME *prme*. Third, we use a function *predict* which, based on *prme* and the to-be-factorized sequence (suffix), suggests a candidate RME for factorization. If the prediction succeeds, the RME is added to the factorization without an index lookup. If the function returns (0,0,0,0), this means that no RME was predicted, and consequently a lookup in the reference indexes needs to be performed. Whether this algorithm is optimal depends on the *predict*-function. We discuss three instantiations of the *predict*-function below.

**Table 2:** Referential factorization with RME prediction.

	**Input:** Sequence database *S* = {*s*_1_,…, *s*_*n*_} and collection of references *REF* = {*ref*_1_,…, *ref*_*m*_}
	**Output:** factorized sequence database *fsd* = {*f*_1_,…, *f*_*n*_}
1:	Let *fsd* = ∅
2:	**for** 1 ≤ *i* ≤ *n* **do**
3:	Let *f* be an empty list
4:	Let *s* = *s*_*i*_
5:	**while** ∣*s*∣ ≠ 0 **do**
6:	Let *candidate* = *predict*(*s*, *prme*, *REF*)
7:	**if** *candidate* ≠ (0,0,0,0) **then**
8:	Add *candidate* to the end of *f*
9:	Remove the first ∣*candidate*∣ symbols from *s*
10:	**else**
11:	Let *pre* be the longest prefix of *s*, such that $(pos,pre)\in search{\left(ref_{i}\right)}_{pre}^{0}$, for a number *pos*, and there exists no 1 ≤ *j* ≤ *n*, with *j* ≠ *i* and *ref*_*j*_ contains a longer prefix of *s* than *ref*_*i*_
12:	**if** *s* ≠ *pre* **then**
13:	Set *rme* = (*ref*_*i*_, *pos*,∣*pre*∣, *s*(∣*pre*∣))
14:	Add *rme* to the end of *f*
15:	Remove the first ∣*pre*∣+1 symbols from *s*
16:	**else**
17:	Set *rme* = (*ref*_*i*_, *pos*,∣*pre*∣ − 1, *s*(∣*pre*∣ − 1))
18:	Add *rme* to the end of *f*
19:	Remove the prefix *pre* from *s*
20:	**end if**
21:	**end if**
22:	Let *prme* = *rme*
23:	**end while**
24:	Add *f* to the end of *fsd*
25:	**end for**

#### 2.1.1 Local matching

A very simple, yet effective, technique is local matching [18]. The idea is as follows: This heuristic tries to find a long RME in the area near the end of the previous RME into the reference. Intuitively, if many sequence deviations consist of SNPs/small indels (with respect to the reference), we often do not need to perform an expensive index lookup in the (large) reference dictionaries, but can find a good next RME by searching left and right to the end of the previous RME. We restrict the maximum distance from the previous match end by a parameter *δ*_*max*_. This exploitation of locality yields fewer cache misses and is efficiently supported by all computer architectures. In addition, we only use the best local matching RME, if it is longer than a threshold *l*_*min*_. The prediction function of local matching is formalized in Table 3. This heuristic significantly increases factorization speed (see Section 5.1 for analysis). This heuristic is, however, not optimal: It might happen that local matching encodes rather short consecutive matches, while longer RMEs could be encoded using a different part of the reference (or even a different reference sequence). This raises the question, whether it is possible to avoid index lookups with the guarantee of optimality.

**Table 3:** Local matching prediction.

	**Input:** Sequence *s*, RME *prme*, and collection of references *REF* = {*ref*_1_,…, *ref*_*m*_}
	**Output:** RME *candidate*
1:	Let *candidate* = (0,0,0,0)
2:	**for** −*δ*_*max*_ ≤ *δ* ≤ −*δ*_*max*_ **do**
3:	Let *p* = *prme*.*pos*+*prme*.*l*+1+*delta*
4:	Let *l* be the length of the longest prefix of *ref*_*prme*.*id*_(*p*,∣*ref*_*prme*.*id*_∣ − *p*) matching with the prefix of *s*
5:	**if** *l* ≥ *candidate*.*l*∧*l* ≥ *l*_*min*_ **then**
6:	Set *candidate* = (*prme*.*id*, *p*, *l*, *s*(*l*))
7:	**end if**
8:	**end for**

#### 2.1.2 Optimal RME prediction based on factorization history

At first, it seems unintuitive that one can avoid index lookups and still guarantee optimality. However, when factorizing a large number of highly-similar sequences, lookups for the same prefix often are performed repeatedly, which lead to the same RME (if the collection of references remains fixed during factorization; this is our assumption here). If such redundant lookups could be avoided, the factorization time can be tremendously reduced. We give an example for redundant lookups:

**Example 2** *We want to factorize the four sequences from Example 1 against *REF*. When we factorize *s*_1_, we start with a longest matching prefix lookup of AACTCGGGCGGTGGCCAG…, and obtain RME* (1,0,8, *C*). *The next three longest matching prefix lookups yield* (0,10,13,*T*), (0,24,11,*A*), *and* (1,32,4,*A*), *respectively. After we factorized *s*_1_ against *REF*, we start with the factorization of *s*_2_. We look up the longest matching prefix of AACTCGGGCGGTGGCCAG… in the references and obtain the RME* (1,0,8,*C*). *This RME is identical to the first RME we obtained during the factorization of *s*_1_ against *REF*, since it describes the identical longest matching prefix. Similarly, the next longest matching prefix lookup yields* (0,10,13,*T*), *which was already used as the second RME in the factorization of *s*_1_. This small example shows that we repeatedly lookup similar sequences and often obtain identical RMEs describing a common prefix of a suffix*.

One possibility to avoid repeated lookups of the same prefix is to keep track of the seen prefixes and the RMEs describing them, in a Patricia-tree-like data structure. However, this approach requires large amounts of main memory, and the tree structure is again subject to many cache-misses during traversal. Intuitively, we would like to have a compressed representation which helps us to predict the next RME, based on the history of factorization so far. For this purpose, we introduce a new data structure called RME graph.

A RME graph describes all consecutive pairs of RMEs for multiple factorizations. The RMEs are modeled as vertexes and there exists an edge between two vertexes, if these two RMEs occur consecutively in any referential factorization of the FSD. We augment the edges in RME graphs with position information (identifier of the referential factorization and offset of the second RME in the pair).

**Definition 3 (RME Graph)** *The* RME Graph *of a factorized sequence database* *fsd* = {*f*_1_,…, *f*_*n*_} *is defined as a graph* 〈*V*, *E*〉, *such that* *V* = {*rme*∣∃*i*.*rme* ∈ *f*_*i*_} *is the set of vertexes and E is a set of edges, such that* (*rme*_1_,(*i*, *pos*), *rme*_2_) ∈ *E* *if and only if we have* *offset*(*f*_*i*_, *rme*_1_) = *pos* − ∣*rme*_1_∣, *offset*(*f*_*i*_, *rme*_2_) = *pos*, *and* *rme*_2_ *is not the last RME in* *f*_*i*_.

Note that we do not store the last RME of each referential factorization. This is necessary in order to guarantee optimality of compressions. We explain this below.

**Fig 2:**
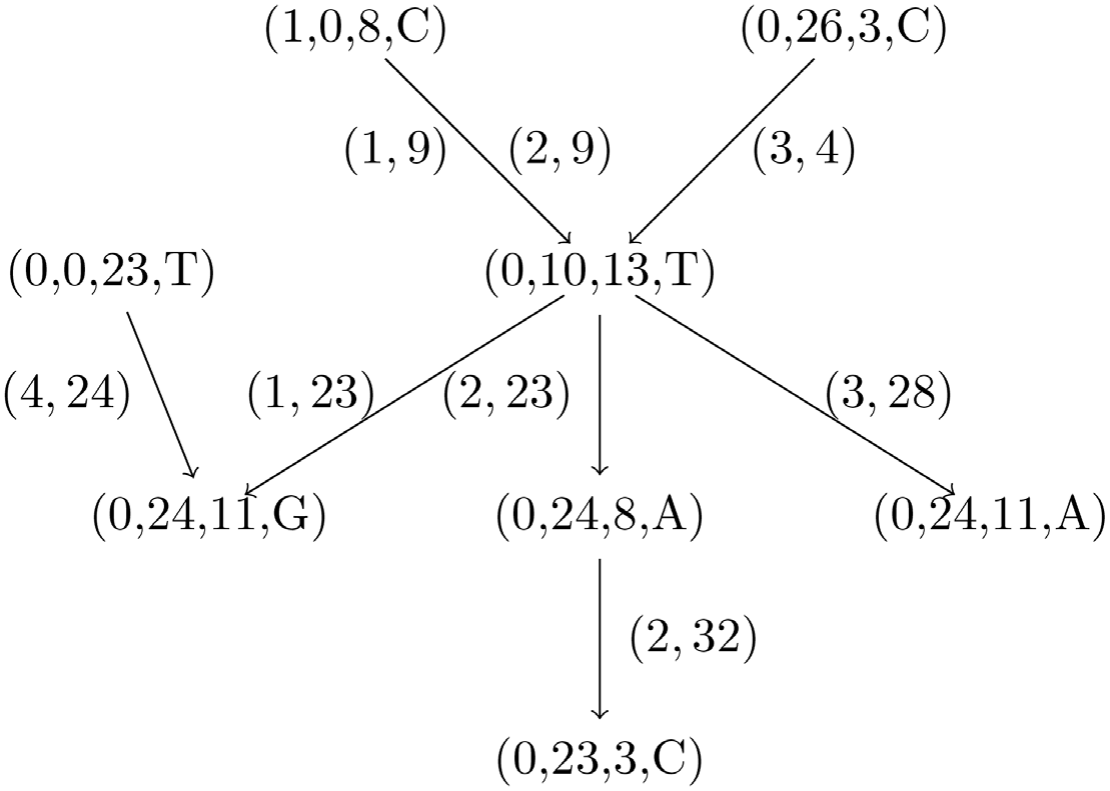
RME graph for the sequences from Example 1. The graph has 8 nodes and 8 edges. The edge from (1,0,8,*C*) to (0,10,13,*T*) occurs two times: one time for the factorization of *s*_1_ and another time for the factorization of *s*_2_.

**Example 3** *The RME graph for the sequences from Example 1 is shown in Fig 2*.

Here we exploit RME graphs to avoid index lookups and still preserve optimality. Suppose that we are factorizing a sequence database {*s*_1_,…, *s*_*n*_} against *REF* and are processing *s*_*i*_, i.e. we have already factorized {*s*_1_,…, *s*_*i* − 1_}. Let *rmeg* = 〈*V*, *E*〉 be the RME graph for {*f*_1_,…, *f*_*i* − 1_}. We define a notion called *RME matching* to decide, whether a RME in a RME graph describes the prefix of a sequence.

**Definition 4 (RME matching)** *A RME* (*id*, *s*, *l*, *m*) matches *a sequence* *s*, *if* *s*(0, *l*+1) = *id*(*s*, *l*) ∘ *m*.

During factorization, if any *rme* = (*id*, *s*, *l*, *m*) in the RME graph matches current *s*_*i*_ suffix, then there cannot be any better (longer) match in the references (see the proof below), and thus, we use *rme* as a compressed representation of the prefix of length *l*+1 of *s*_*i*_. This prediction function is presented in Table 4. Note that this prediction function takes an additional argument: The RME graph of the sequences factorized before, i.e., if the algorithm from Table 2 is currently factorizing *s*_*i*_, then the RME graph *rmeg* is built over the factorizations of *s*_1_ to *s*_*i* − 1_.

**Table 4:** General RME graph prediction.

	**Input:** Sequence *s*, RME *prme*, a collection of references *REF* = {*ref*_1_,…, *ref*_*m*_}, and a RME graph *rmeg* = 〈*V*, *E*〉
	**Output:** RME *candidate*
1:	Let *candidate* = (0,0,0,0)
2:	**for** *rme* ∈ *V* **do**
3:	**if** *rme* matches s **then**
4:	Set *candidate* = *rme*
5:	**end if**
6:	**end for**

**Proposition 3** *The algorithm from Table 2 with the prediction function of Table 4 computes a minimal factorized sequence database for* *S* *and* *REF*.

**Proof 3** *We prove Proposition 3 by induction on the number of sequences in* *S*. *The induction base is* ∣*S*∣ = 1, *such that the RME graph is empty. In this case, the algorithm is identical to Table 1 (for multiple sequences), and thus, computes an optimal factorization. The induction step is as follows: Suppose we have factorized* *n* − 1 *sequences from* *S* *and factorize* *s*_*n*_. *The interesting case is that we find a RME* (*ref*, *pos*, *l*, *m*) ∈ *V*, *i.e., the nodes of the RME graph, which matches* *s*_*n*_, *as defined in Definition 4. We need to show that there is no other RME into* *REF* *whose length is greater than* *l*+1. *Assume on contrary that there exists one such RME* (*ref*_*X*_, *pos*_*X*_, *l*_*X*_, *m*_*X*_) *with* *l*_*X*_ > *l*. *This implies that at least the first* *l*+1 *characters of reference* *ref*_*X*_ *starting from position* *pos*_*X*_ *match with* *s*_*n*_. *By induction hypothesis, all RMEs in* *rmeg* *describe maximal matches with respect to* *REF*. *But, if* (*ref*, *pos*, *l*, *m*) *matches* *s* *and* (*ref*_*X*_, *pos*_*X*_, *l*_*X*_, *m*_*X*_) *matches* *s* *as well, then* (*ref*, *pos*, *l*, *m*) *is not the longest match in the references, since we assume that* *l* < *l*_*X*_.

Note that for Proposition 3 to hold, we cannot store the last RME of a factorization in the RME graph, since these RMEs do not necessarily describe a longest match (see Line 17–19 in Table 2). Thus we omit the last RME of each factorization from RME graphs. To further support the understanding of Proposition 3 and its corresponding proof, we provide a small example:

**Example 4** *Revisiting Example 2, assume that we have factorized* *s*_1_ *and are now factorizing* *s*_2_. *The first RME is* (1,0,8,*C*). *Next, we try to factorize the suffix GGTGGCCAGGCGGT… of* *s*_2_, *which is matched by the RME* (0,10,13,*T*). *This RME was added to the RME graph during the factorization of* *s*_1_. *Now suppose that there would exist a RME into the reference longer than 13 symbols. In this case, the subsequence GGTGGCCAGGCGGT must occur somewhere in one of the references. However, if this subsequence occurred in the references, then we would have already factorized* *s*_1_ *differently when obtaining* (0,10,13,*T*), *since always searched for the longest possible matches*.

We described how to avoid costly index lookups in the references, while guaranteeing an optimal factorization. The major shortcoming of the algorithm in Table 2 using the prediction function of Table 4 is that it checks for *all* RMEs in *rmeg*, whether they match with a to-be-factorized prefix. If the number of RMEs in the RME graph grows, checking all RMEs can be more time-consuming than a single longest matching prefix-lookup in all references. Moreover, the vast majority of these checks eventually fail, since only one/few previously encoded RMEs match a current prefix. We need a strategy to select a *promising* subset of all RMEs in *rmeg* to reduce the number of failed RME matching checks. We introduce two such heuristics below.

We would like to select a subset of RME candidates based on the current context of factorization. A simple, yet very effective, strategy is to exploit the previously encoded RME as a context of factorization, just as we did with local matching: Identical RMEs are often followed by similar/identical RMEs. All we need to do is to look up the previously encoded RME *prme* in the RME graph, get all successor RMEs, i.e. all RMEs which followed *prme* in previously factorized sequences, and check for these RMEs whether they match a current prefix of the to-be-factorized sequence. It is important to note that reducing the number of candidates still preserves the factorization algorithm optimal, since in the worst case an index lookup is performed in all references (just that we might miss some RMEs, whose lookup could have been avoided). We call this strategy *SUCC* (for successor). The *predict*-function is shown in Table 5.

**Table 5:** RME graph successor prediction (SUCC).

	**Input:** Sequence *s*, RME *prme*, a collection of references *REF* = {*ref*_1_,…, *ref*_*m*_}, and a RME graph *rmeg* = 〈*V*, *E*〉
	**Output:** RME *candidate*
1:	Let *candidate* = (0,0,0,0)
2:	**for** (*prme*,(*i*, *pos*), *rme*) ∈ *E* **do**
3:	**if** *rme* matches s **then**
4:	Set *candidate* = *rme*
5:	**end if**
6:	**end for**

Only checking RMEs which have exactly the same predecessor RME can be too strict. We propose a more relaxed heuristic which exploits the idea behind local matching. The intuition is that RMEs which end at the same position in the reference often have the same next RME, if the unfolded sequences are highly similar. The algorithm is shown in Table 6. This heuristic keeps the factorization algorithm optimal, since in the worst case an index lookup is performed in all references. We call this strategy *POSI* (for position).

**Table 6:** RME graph position prediction (POSI).

	**Input:** Sequence s, RME *prme*, a collection of references *REF* = {*ref*_1_,…, *ref*_*m*_}, and a RME graph *rmeg* = 〈*V*, *E*〉
	**Output:** RME *candidate*
1:	Let *candidate* = (0,0,0,0)
2:	**for** *rme* ∈ *V*, such that there exists a *rme*_*t*_ ∈ *V* with (*rme*_*t*_,(*prme*.*ref*_*id*_, *prme*.*s*+*prme*.*l*+1), *rme*) ∈ *E* **do**
3:	**if** *rme* matches s **then**
4:	Set *candidate* = *rme*
5:	**end if**
6:	**end for**

### 2.2 Decreasing memory usage

The amount of memory required for factorization against multiple references grows linearly with the number of references: Due to resource constraints, (optimal) referential factorization against many, large references is only feasible on high-end systems. For instance, a compressed suffix tree for a single whole human genome takes approx. 6 GB, while an enhanced suffix array consumes 30 GB and more.

Note that we have proposed a heuristic for multi-reference factorization [18]. The general idea is that instead of factorizing against indexed uncompressed references, we also factorize sequences against factorized references, exploiting the fact that identical subsequences are often encoded with identical RMEs. First, given a set of references *REF* = {*ref*_1_,…, *ref*_*m*_} all references are factorized against a single reference (*ref*_1_) only. Second, all to-be-factorized sequences in a sequence database *S* are factorized against *ref*_1_, using, for instance, the algorithm from Table 1. At this stage, we have a set of factorized references and a set of factorized sequences. In the final step, all factorized sequences are rewritten against the factorized references, which eventually yields a multi-reference factorization. A description of this algorithm is shown in Table 7. We refer to this algorithm as *reference extension*, since starting from one reference, a factorization is extended to multiple references.

**Table 7:** Referential factorization with reference extension.

	**Input:** Sequence database *S* = {*s*_1_,…, *s*_*n*_} and collection of references *REF* = {*ref*_1_,…, *ref*_*m*_}
	**Output:** factorized sequence database *fsd* = {*f*_1_,…, *f*_*n*_}
1:	Factorize *REF* against {*ref*_1_} and obtain *fsd*_*ref*_ (using the algorithm from Table 2)
2:	Let *rmeg* be the RME graph of *fsd*_*ref*_
3:	Factorize *S* against {*ref*_1_} and obtain *fsd*_*S*_ (using the same algorithm as for *fsd*_*ref*_
4:	Let *fsd* = ∅
5:	**for** *f*_*i*_ ∈ *fsd*_*S*_ **do**
6:	Let *fo* = ∅
7:	**while** ∣*f*_*i*_∣ ≠ 0 **do**
8:	Extend *f*_*i*_ to *fo*, using *rmeg* and *fsd*_*ref*_
9:	**end while**
10:	Add *fo* to *fsd*
11:	**end for**

The actual reference extension is encoded in Line 8 of Table 7, here only an abstract description of the extension process is shown. In [18], the extension process was implemented as follows: Traversing the RMEs of *f*_*i*_ from left to right, greedily find the longest matches in *fsd*_*ref*_ The main trick is to view RMEs as symbols, which can be identical, and similarly to the algorithm from Table 1, we can encode lists of RMEs as pointers into other referential factorizations. If a longest match is found that covers at least two RMEs in *f*_*i*_, then this subsequence of RMEs in *f*_*i*_ is replaced with a single RME into the (unfolded) reference. We refer to this process as *right-extension*, since the RMEs in a factorized sequence are traversed from left to right. Here, we exploit RME graphs for extension, since, given two identical RME, we can find the right-hand RMEs by visiting the successors of the identical RMEs.

However, further analysis of reference extension showed that changing the direction of extension to right-to-left, can actually decrease the number of RMEs. We show an example explaining why left-extension is often more effective.

**Example 5** *We are given one reference and two to-be-factorized sequences*:

$$\begin{array}{cc} ref_{1}= & AAGCTCGGGAGGTGGCCAGGCGGCAGGAAGGCGCACCC\\ s_{1}= & TAGGAGCTCGGGAGGGCCAGGCGGCAGGAAGGCGCACCC\\ s_{2}= & ATAGGAGCTCGGGAGGGCCAGGCGGCAGGAAGGCGCACCC \end{array}$$

*Note that* *s*_2_ = *A* ∘ *s*_1_. *We obtain the following referential factorizations*:

$$\begin{array}{cc} f_{1}=fact\left(s_{1},\left\{ref_{1}\right\}\right)= & \left[(0,4,1,A),(0,7,4,C),(0,4,8,G),(0,15,22,C)\right]\\ f_{2}=fact\left(s_{2},\left\{ref_{1}\right\}\right)= & \left[(0,0,1,T),(0,24,4,G),(0,3,9,G),(0,15,22,C)\right] \end{array}$$

*Suppose that in addition we want to exploit similarities between* *f*_2_ *and* *f*_1_, *by referentially factorizing* *f*_2_ *against (factorized)* *f*_1_. *If we use right-extension (see above), the two sequences only share one RME:* (0,15,22,*C*). *This RME is the right-most RME in* *f*_2_, *and thus, right extension will not rewrite* *f*_2_ *at all: The problem is that the commonalities between* *s*_1_ *and* *s*_2_ *are only identified after two identical RMEs encoding identical subsequences. In fact, the last two RMEs of* *f*_1_, [(0,4,8,*G*),(0,15,22,*C*)], *describe a suffix of* [(0,3,9,*G*),(0,15,22,*C*)]. *The same observation holds when further extending to the left:* [(0,7,4,*C*),(0,4,8,*G*),(0,15,22,*C*)] *is a suffix of* [(0,24,4,*G*),(0,3,9,*G*),(0,15,22,*C*)] *although both sequences are encoded with distinct RMEs. Extending further to the left, we find the* *f*_1_ *is a subsequence of* *f*_2_ *and that the last three RMEs of* *f*_2_ *encode a subsequence of* *f*_1_. *These three RMEs in* *f*_2_ *can be replaced with* (1,0,36,*C*), *where 1 is the identifier for factorized reference* *f*_1_.

We exploit the observation from Example 5 as follows: Given two identical RMEs as anchors, one in a factorized reference and one in a to-be-extended factorized sequence, we unfold the RMEs on-the-fly towards the left, until neither of the two unfolded sequences is subsequence of the other one. We find the longest possible extension (among all identical RMEs in all references) and rewrite the RME-subsequence of *f*_*i*_ accordingly to represent the longer match into the factorized reference. We call this process *left-extension*. In general, it is possible to combine left-extension with right-extension. However, the design of such algorithms is complicated, since one needs to factorize in both directions and possibly repeatedly replace previously factorized subsequences.

## 3 Results

We used six real-world datasets in our experiments: 110 sequences from two different chromosomes of Arabidopsis thaliana (AT1 and AT5), 110 sequences from three human chromosome (HG1, HG10, HG21), and a set of 38 yeast genomes (yeast). Details of the datasets are shown in Table 8. Human chromosomes have a very high sequence similarity among different individuals and excellent compression rates have been shown in the literature [8–10]. We selected three distinct chromosomes to cover different lengths. Yeast genomes are the most dissimilar sequences in our dataset and compression rate is expected to be much worse than for Human chromosomes. The degree of similarity between Arabidopsis thaliana chromosomes is in between Human chromosomes and yeast genomes.

**Table 8:** Datasets for evaluation.

Name	Description	No. of References	No. of sequences	Avg length	∣Σ∣
AT1	1st chromosome of Arabidobsis thaliana	Up to 10	100	30,427,671	5
AT5	5th chromosome of Arabidobsis thaliana	Up to 10	100	26,975,502	5
HG1	1st Human chromosome (longest)	Up to 10	100	249,184,427	5
HG10	10th Human chromosome (average length)	Up to 10	100	135,493,299	5
HG21	21st Human chromosome (smallest)	Up to 10	100	48,113,871	5
yeast	Complete yeast genomes	Up to 10	28	12,592,856	5

We used the same datasets as in [18]. The data can be obtained as follows: Human genomes can be found in the archive ftp://ftp.1000genomes.ebi.ac.uk/vol1/ftp/release/20110521/ of the 1000 Genomes Project [1] as VCF [27] files. Since the VCFs contain phased genome data, two haploid sequences can be derived for each individual. In our experiments, we have only extracted one consensus sequence for each individual, always choosing the first sequence only. For instance, variant rs73877820 of chr22 contains (among others) the following two data fields: a) 1∣0:1.000: −5.00,0.00, −5.00 and b) 0∣1:0.900: −2.49, −0.46, −0.19. In the former case (a), we incorporated rs73877820 into the consensus sequence, while in the latter case (b), we chose the value of the reference sequence instead. The data for Arabidopsis thaliana is taken from the 1001 Genomes project [28] from release GMINordborg2010 at http://1001genomes.org/data/GMI/GMINordborg2010/releases/current/. For each strain, a file with SNPs with respect to the reference TAIR9 is provided. The dataset for yeast [29] genomes is available as well at ftp://ftp.sanger.ac.uk/pub/users/dmc/yeast/latest/cere_assemblies.tgz.

We conducted an extensive set of experiments for the evaluation of factorization methods and heuristics, in terms of factorization size, speed, and main memory usage. All experiments were run in a server with 1 TB RAM and 4 Intel Xeon E7-4870, where all experiments were run in a single thread only. Parallelization is left for future work. Our analysis shows that single-threaded factorization algorithms already face an IO-bottleneck for highly-similar sequences. Code was implemented in C++, using the BOOST library, CST [30] for compressed suffix trees, and SeqAn [31] for enhanced suffix arrays. Our code can be downloaded at http://www.informatik.hu-berlin.de/~wandelt/MRF for free academic use.

The setup for our experiments is described in Section 3.1 and parameters for experiments are analyzed in Section 5.1 In Section 3.2 we report on the results of optimal multi-reference factorization and discuss heuristic factorization in Section 3.3. We analyze the speed of multi-reference factorization techniques in Section 3.4 and report on main memory usage in Section 3.5.

### 3.1 Experimental Setup

We compared 30 configurations: For each configuration we chose to instantiate the following: the type of index structure for reference sequences, the match finding algorithm, and the reference extension method. These components of a configuration are described as follows:

- We have used three index structures for references: Compressed suffix trees (**CST**), enhanced suffix array (**ESA**), and a *k*-mer index (**KMER**). The value of *k* is chosen in Section 5.1.
- We compared four match finding algorithms: Basic greedy lookup in the references (**BASE**), RME-successor prediction (**SUCC**), positional RME prediction (**POSI**) and local match-finding (**LOMA**). The parameters for LOMA are chosen in Section 5.1.
- We distinguished three types of reference extension methods: Left-extension (**L**), right-extension (**R**) and no extension (**N**, where all references are indexed in main memory).

A configuration is denoted as INDEX_MATCHFINDING_EXTENSION, for instance, CST_POSI_N is a configuration which creates a compressed suffix tree (CST) for each reference (N) and uses positional RME prediction (POSI). We checked all combinations except from *KMER*_*SUCC* and *KMER*_*POSI*, since *k*-mer-based indexes are not applicable for optimal factorization. The following configurations compute an optimal factorization: CST_BASE_N, CST_POSI_N, CST_SUCC_N, ESA_BASE_N, ESA_POSI_N, and ESA_SUCC_N. All other configurations compute a factorization without optimality guarantee.

First, we chose the following parameters for indexing and match finding techniques: The choice of *k* for a *k*-mer index is set to 16, *δ* = 10, and *l*_*min*_ = *k* = 16 (see Appendix for rationale/experiments).

### 3.2 Optimal Factorization

**Fig 3:**
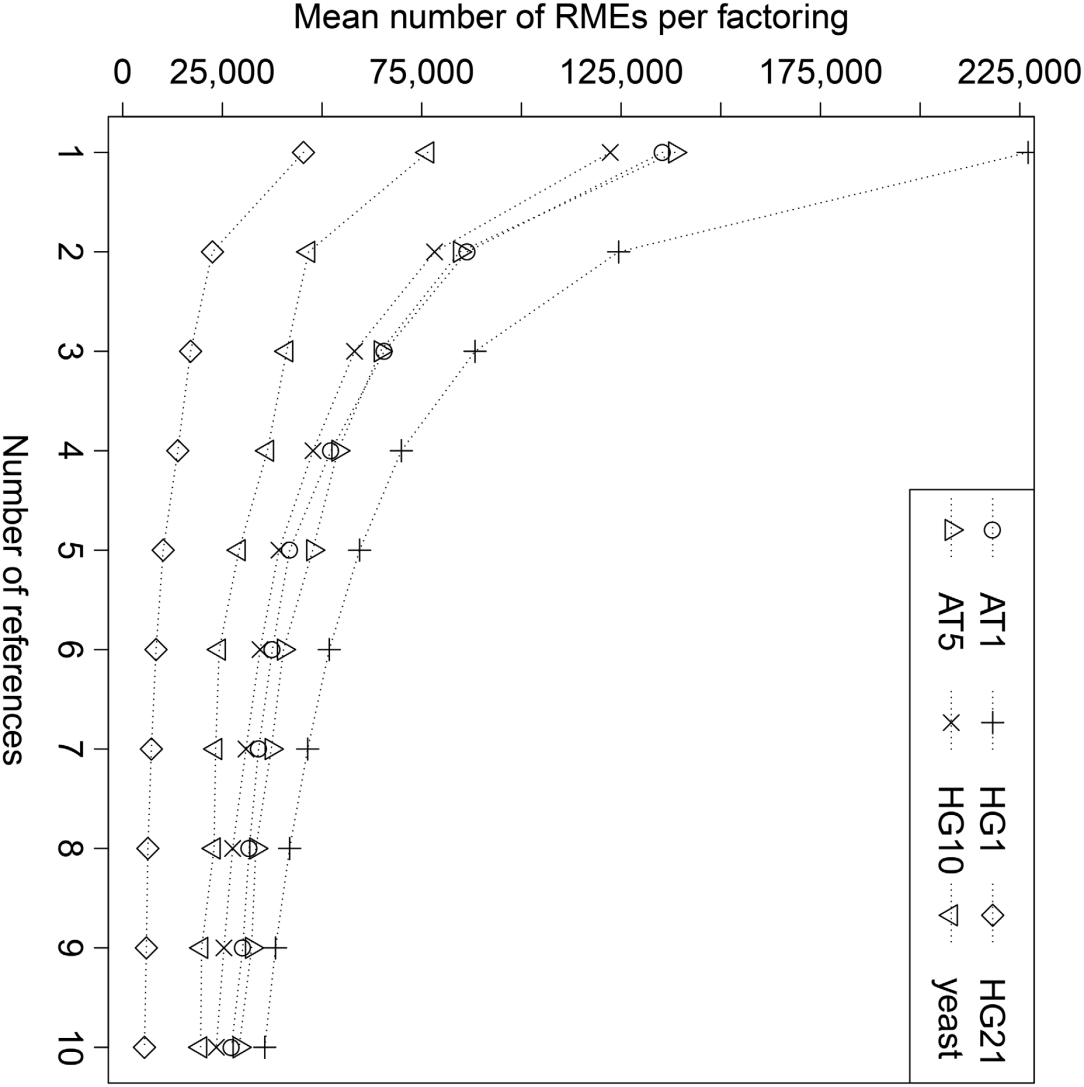
Optimal (average) number of RMEs as computed by ESA_BASE_N. With an increasing number of references, the number of RMEs for encoding decreases significantly.

We compared the size of optimal factorization (in number of RMEs) for a varying number of references. We repeated the experiments with different randomly selected references and report the average values. The references were external to the datasets we used, i.e., references and to-be-factorized sequences were disjoint. The results are shown in Fig 3. For all six datasets, the average size of factorizations is reduced with an increasing number of references. In fact, one can fit each of the curves with a power-law function accurately, with very high R2. The result is as follows:

- AT1: 137,663.2*x ^− 0.7073^, *R* ^2^ = 0.9968
- AT5: 136,365.9*x ^− 0.6681^, *R* ^2^ = 0.9987
- HG1: 218,538.7*x ^− 0.7990^, *R* ^2^ = 0.9984
- HG10: 126,679.0*x ^− 0.7265^, *R* ^2^ = 0.9986
- HG21: 45,474.3*x ^− 0.9297^, *R* ^2^ = 0.9955
- yeast: 75,784.7*x ^− 0.5953^, *R* ^2^ = 0.9860

Sequences from Human genomes have the highest absolute exponents, which means that additional references can be much better exploited than for AT and yeast. The R2-value for all six datasets is very high. Our evaluation suggests that the number of RMEs can be approximately predicted given the species/type of a sequence and a number of references. This finding should be further investigated in future research. Previous research [18] showed that 60 references (using heuristic factorization) can reduce the size of compressions for Human genomes by 80% and more. Our experiments show that using optimal factorization already 6–10 references suffice to decrease the number of RMEs by 80%. For instance, HG21 sequences are reduced from 45,341 RMEs (on average, with one reference) to 5,464 RMEs (on average with ten references), a reduction by 88%. Similarly, for AT the average number of RMEs is reduced from 135,376 to 27,284, a reduction by 80%. Note that the compressibility of datasets cannot be compared for different datasets in Fig 3, since one needs to take into account the length of the sequences, e.g., yeast sequences in our dataset are 20 times shorter than HG1 sequences. Thus, although the curve for yeast is located below four other curves, the factorization rate of yeast is worse (when taking into account length of sequences as well).

### 3.3 Heuristical Factorization

**Fig 4:**
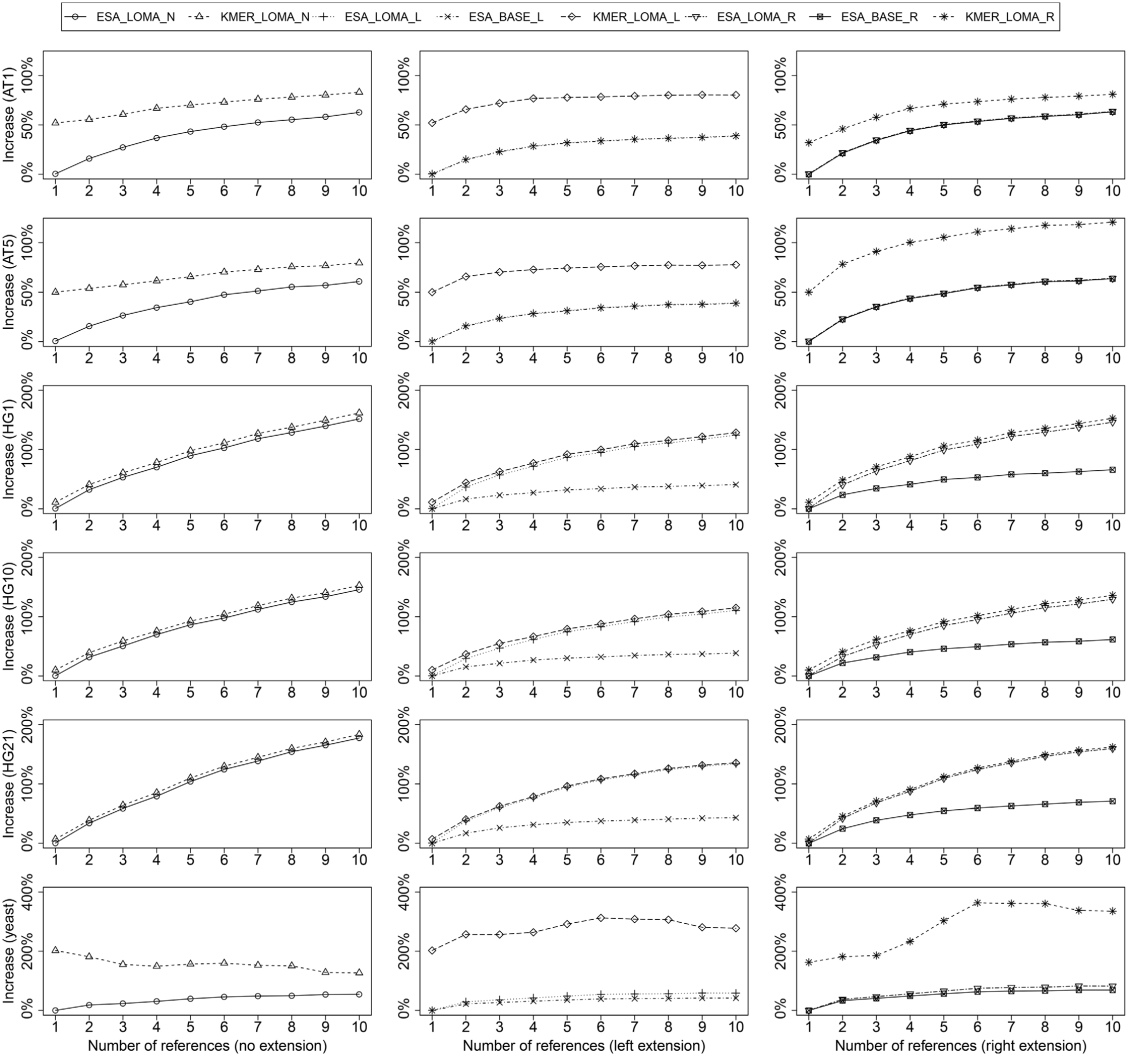
Increase in the number of RMEs for approximate factorization techniques: without extension (left), with left-extension (center), and with right-extension(right). With an increasing number or references, all techniques gradually deviate from the optimum, up to 100% and more. Left-extension is always more close to the optimum than right-extension. Extensions built on BASE are more efficient than the ones built on LOMA.

We evaluated the quality of factorization heuristics compared to the optimal factorization. The increase in number of RMEs of each heuristic against the optimal factorization as a baseline is shown in Fig 4. We summarize main insights as follows:

1. ESA-based local matching is always better than KMER-based local matching: This is explained as follows: if local-matching does not yield a match, then an optimal match is found with ESA. KMER only can find approximate matches, if the match length is shorter than *k*. For all HG, average match lengths are larger than *k* and thus often an optimal match is found. For the other species (AT and yeast), matches are often rather short and a RME consists of only one character, if no match longer/equal than *k* can be found.
2. Left/right-extension on an optimal factorization creates smaller representations than factorization based on local matching. The explanation is as follows: When using local matching for initial factorization, the algorithm is sometimes stuck in finding short local matches, while larger matches could be found with an index. These short matches are then encoded with distinct RMEs for different factorizations and thus do not provide a starting point for extension. An optimal factorization, on the other hand, produces more often identical RMEs for the same subsequence, which leads to more starting points for left/right-extension.
3. Left-extension often creates fewer RMEs than right-extension. The reason is as follows: factorization with locality-based heuristics prefers local matches over (possibly better) matches more far away. Thus, two identical subsequences from two different sequences are sometimes encoded with different RMEs, if factorization starts in different contexts. Only once a global (large) match is found, the remaining suffixes of the two identical subsequences are encoded with the same RME(s), since the factorization context is equal for the suffixes. Thus, left-extension can uncover new kinds of sequence equalities which are hidden from right-extension.
4. Left/right extension can even lead to (slightly) smaller representations than factorization without extension but local matching. This fact is explained as follows: When using local matching with many reference indexes, the factorizations is sometimes stuck in one reference, while much longer matches could be found in another reference. If, on the other hand, only one reference with extension is used the identical subsequences are partly identified during the extension phase. This observation is very surprising and interesting, since we will show below that factorization with extension needs less memory than factorization without extension: We obtain a better factorization, while always using less memory for references.

### 3.4 Factorization Speed

**Fig 5:**
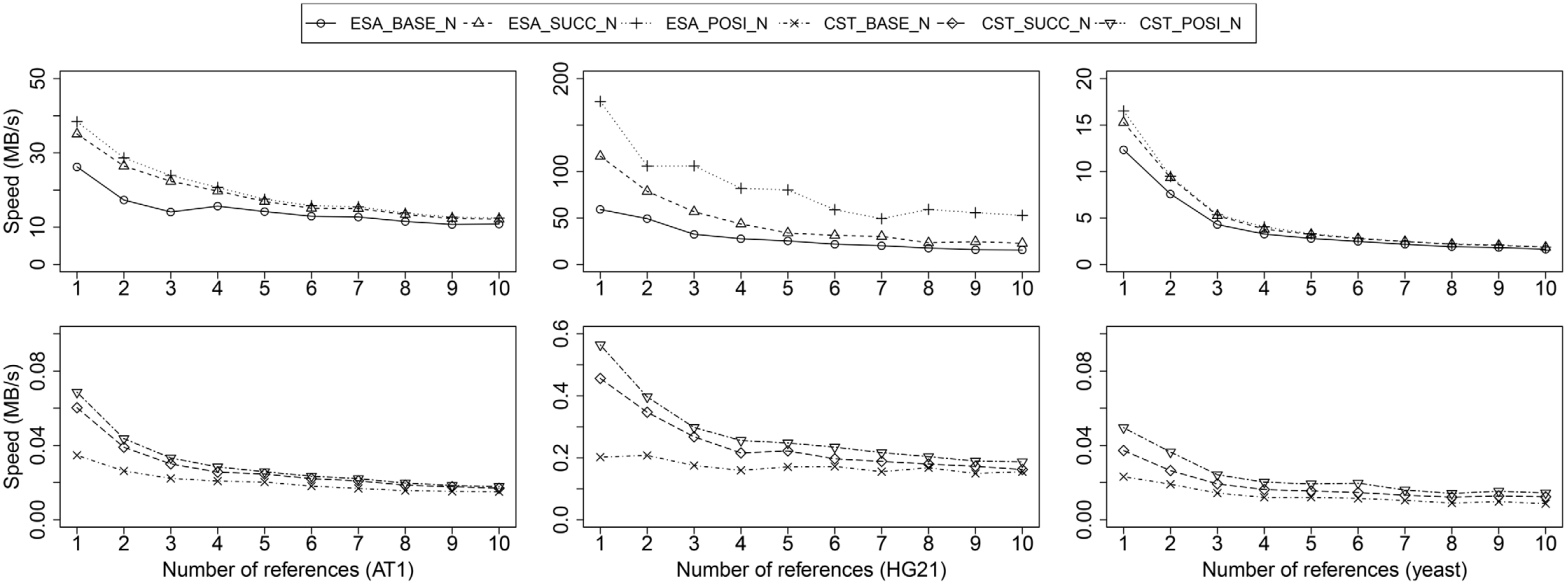
Factorization speed of optimal factorization techniques: based on ESA (top) and based on CST (bottom). The factorization speed significantly, but non-linearly reduces with an increasing number of references. Factorization with ESA index is more than two orders of magnitude faster than CST-based techniques.

We analyzed the factorization speed of selected configurations. First, we evaluated the factorization speed of optimal factorization techniques. The result for AT1, HG21, and yeast is shown in Fig 5. We do not show the results for AT5, HG1, and HG10, since factorization speed within a species is quite stable. The fastest factorization is obtained for HG21 (up to 170MB/s), since a single lookup often finds a long match in the reference sequence(s). Factorizing AT1 is already slower by a factor of four and yeast again two times slower.

**Fig 6:**
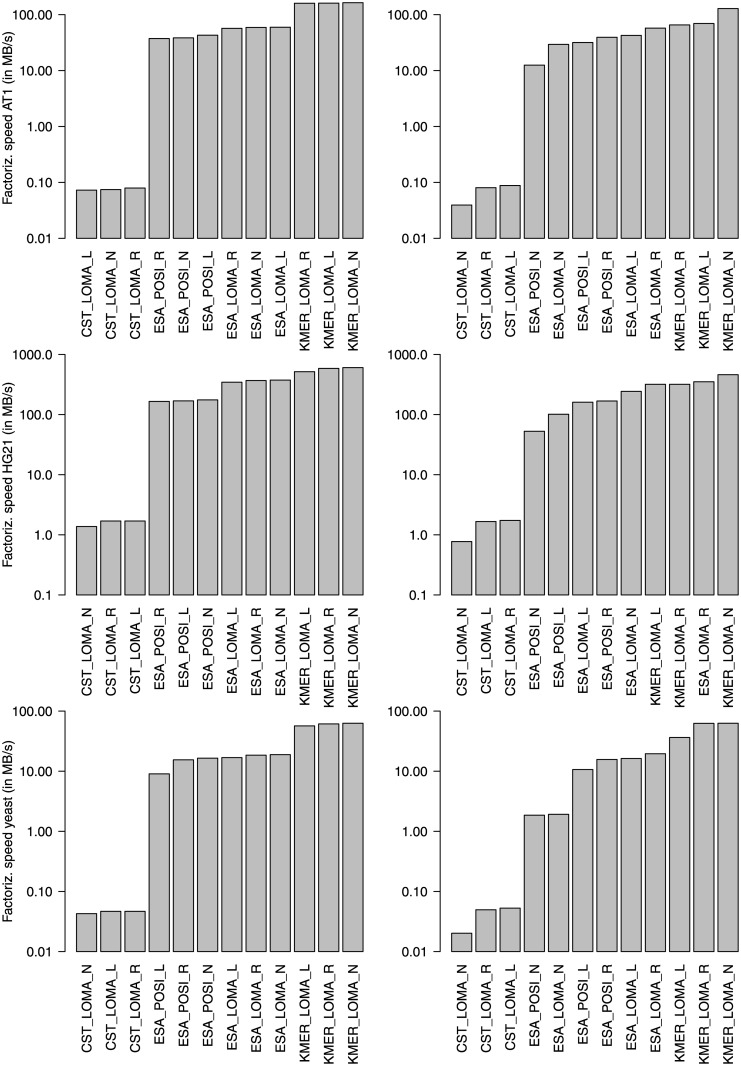
Factorization speed of selected approximate configurations with one reference (left) and ten references (right). More references do not degrade factorization speed as much as in optimal factorization. Local matching on a KMER-index provides the highest factorization speeds for all datasets.

Factorization speed slowly decreases with an increasing number of references, since for each suffix, more lookups have to be performed. At the same time, the average match length increases (see our results in Fig 3), which means that less lookups need to be performed overall. This increase in match length causes non-linear curves for factorization speed in Fig 6: Optimal factorization against 10 references is on average only four times slower than optimal compression against a single reference.

Optimal factorization with ESA is orders of magnitude faster than using a compressed suffix tree. For instance, while HG21 sequences can be compressed at up to 170 MB/s, optimal compression with CST tops at 0.6 MB/s. The reason is that a single index lookup of a prefix takes much longer with a compressed data structure (CST).

The efficiency of our RME graph-based optimizations can be seen for all datasets: With increasing similarity of sequences, SUCC and POSI perform increasingly better (speedup of approx. 30% for yeast and approx. 300% for HG21). Note that, as anticipated, POSI is always faster than SUCC, since more often the optimal match is predicted based on the context of factorization.

We analyzed the factorization speed of selected approximate methods next. The results are shown in Fig 6. Again, CST-based approaches are 2–3 orders of magnitude slower than their ESA/KMER-counterparts. Similarly to optimal factorization, the highest factorization speed is obtained for highly-similar sequences of HG21: up to 604 MB/s for KMER_LOMA_N, i.e., a k-mer-based index with local matching and all references in memory. ESA_LOMA_N, and CST_LOMA_L/R are ranked second and third. Our experiments show that factorization speeds of at least 60 MB/s are possible for each dataset. With an increasing number of references, the factorization speed is gradually reduced, however not as significantly as during optimal factorization.

### 3.5 Memory Footprint

**Fig 7:**
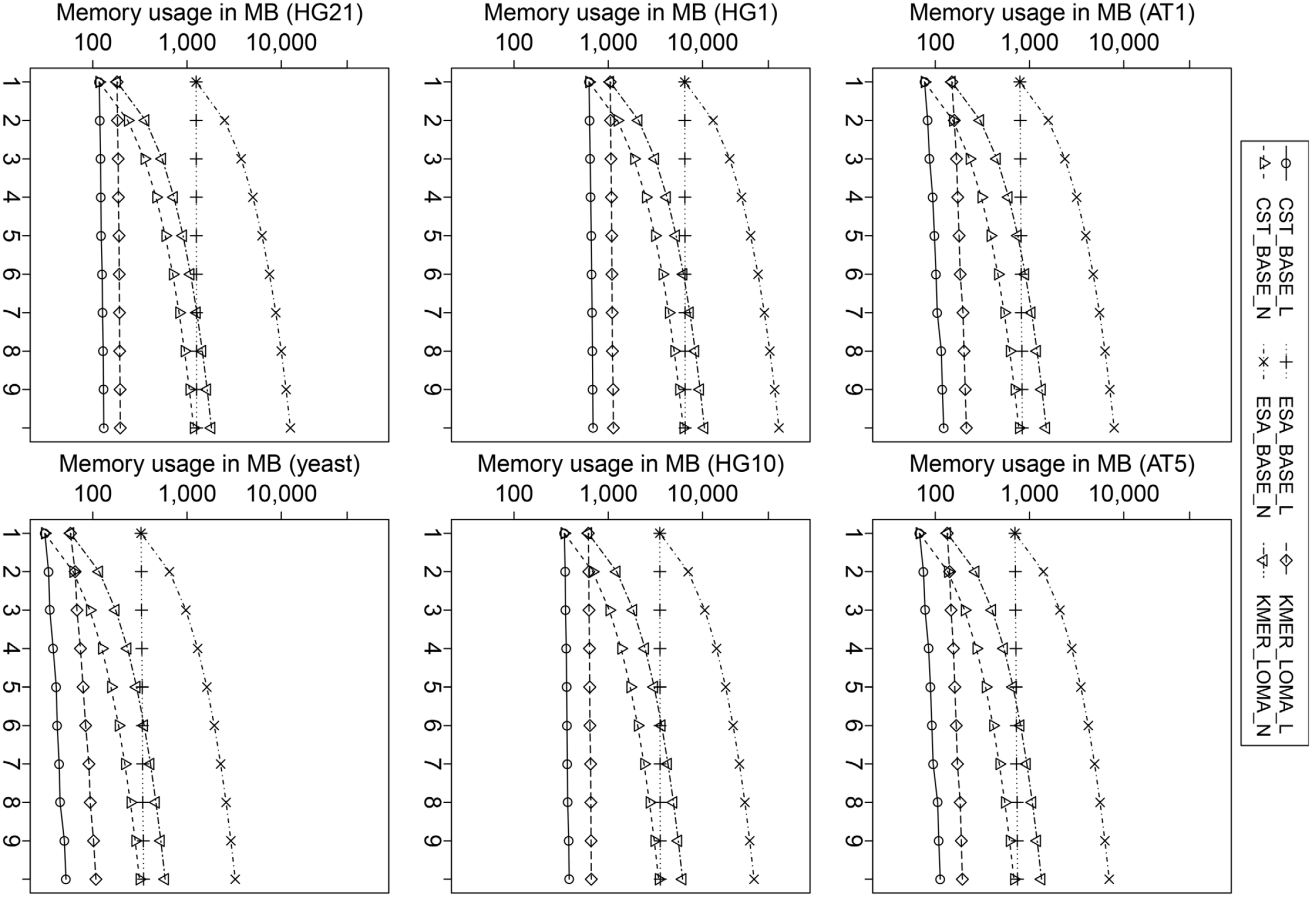
Memory usage of selected configurations. The memory usage without extension is for 10 references up to almost one order higher than for extension-based configurations. ESA requires most memory, CST least, and KMER is in between.

We analyzed the main memory usage of configurations and results are shown in Fig 7. Without left/right-extension, techniques based on CST use the smallest amount of memory, at the price of long factorization time. The reason is that compressed suffix trees store the labels of tree links in a compressed representation, which tremendously reduces the storage, but makes traversing the tree slow, compared to other indexes. In contrast, techniques based on ESA, an enhanced suffix array, use the largest amount of main memory. Accessing suffix arrays is orders of magnitudes faster than CST, since no time is spent on decompression (of the index structure). KMER-based techniques have a memory footprint in between CST and ESA.

With an increasing number of references, configurations with left-extension have a much lower memory footprint than their counterparts with no extension. This shows that additional overhead of RME graphs for references is small compared to the size of an index over unfolded references. The smallest overall memory footprint is obtained by CST_BASE_L. Note that all right-extension techniques use the same amount of memory as their left-extension counterparts, because they work on the same data structure (RME graphs).

It is interesting to note that the ranking of techniques according to the used main memory is the same for all datasets. This observation suggests that the main memory usage of the configurations is rather independent on the degree of similarity between the sequences, but dominated by the number and length of the references. However, it can be seen that configurations with left-extension are sensitive to the similarity of the sequence, e.g., the growth of main memory usage is much steeper for (not so similar) yeast sequences compared to (highly-similar) human sequences: Dissimilar sequences yield many (short) referential match entries, which needs to be kept in the RME graph for reference extension.

## 4 Discussion

### 4.1 Related Work

**Fig 8:**
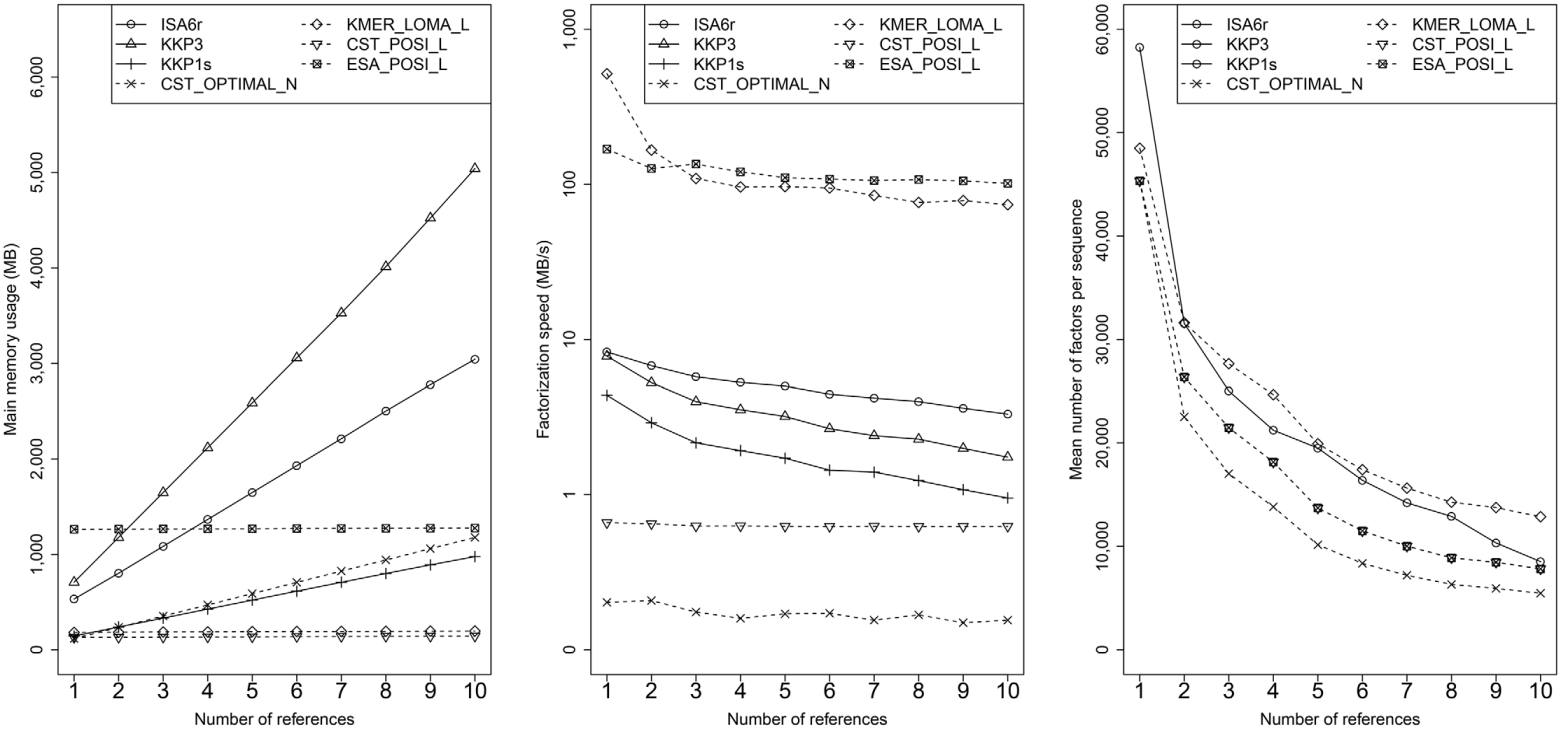
Related factorization algorithms. We show results for related factorization algorithms: main memory usage, factorization speed, and mean number of factors per sequence for dataset HG21. For ease of comparison, we added few interesting instances of our own factorization algorithms (marked with a dashed line). Note that the mean number of factors cannot be compared directly between related work and our algorithms, since our RMEs contain mismatch characters, while related work factorizations are solely pointer into references (which yields more factors, albeit being optimal).

We evaluated experimentally the following other factorization algorithms: ISA6r [23], KKP3 [32], and KKP1s [32], whose implementations are available online at https://www.cs.helsinki.fi/group/pads/lz77.html. According to that webpage, ISA6r is specialized for highly repetitive inputs, KKP3 is the fastest for non-highly repetitive inputs, and KKP1s streams a suffix array from the disk. The experimental results are reported in Fig 8. All factorization algorithms need at least n*∣*LR*∣ bytes of storage in memory (usually more), where *LR* is the length of the shortest reference and *n* is the number of references. Moreover, the factorization speed is often much slower than our own baseline implementation with enhanced suffix arrays. The size of factorizations decreases tremendously with the number of references, similarly to our own experiments.

Sequence compression algorithms caught a lot of attention in the research community during the last years (see [8–10] for broad surveys). In particular, referential, or reference-based, compression algorithms emerged recently [11, 13, 18, 25, 33], outperforming other types of compression algorithms by orders of magnitude, in terms of compression ratio when applied to highly-similar sequences. Similar to dictionary-based techniques [34, 35], these algorithms replace long subsequences of the to-be-compressed input with pointers into an external sequence, which is not part of the to-be-compressed input data [11]. Thus, the reference in referential compression is usually static and defined offline, while dictionaries in dictionary-based compression are built only at compression time. A wide range of compression rates has been reported for reference-based encoding. Given an adequate reference sequence, i.e. a highly-similar sequence, compression rates of 1,000:1 and better are possible, for instance, for human genomes [11, 13, 18, 25, 33]. Another approach to referential sequence compression is based on greedy alignment [36].

We discuss four referential compression techniques in detail: In [25], RLZ, an approach based on self-indexing is described. It works as follows: the algorithm compresses input sequences with LZ77 encoding relative to the suffix-array of a reference sequence. Raw sequences are never stored; even very short matches to the reference are encoded, which makes the method impractical. In [33], RLZopt is presented as an extension of RLZ. The key aspect is longest increasing subsequence computation that allows to efficiently encode positions. It incorporates several improvements, including local look-ahead optimization. A LZ77-style compression scheme, called GDC, based on RLZopt was proposed in [13]. The main difference is that a way for encoding approximate matches is introduced. Also, the Lempel-Ziv parsing scheme originally based on hashing is slightly altered in that the algorithm considers trade-offs between the length of matches and distance between matches. Compression is performed on input blocks with shared Huffman codes, enabling random access. Furthermore, GDC can be started in mode *ultra*, which enables compression against multiple references. FRESCO [18] is a framework for referential sequence compression. The reference sequence is indexed with a 34-mer index and additional heuristics for fast and compact compression are introduced. All the above methods, in general, compress against a single reference only, with some exceptions:

- GDC compresses against a main reference and appends *hard-to-compress* subsequences to the reference, particularly in mode GDC-ultra.
- FRESCO exploits different heuristics for reference selection and reference rewriting. In addition, a proof-of-concept for referential compression against compressed references is proposed, but without an estimation of optimality. Furthermore, the evaluation of FRESCO provided very limited analysis of compression ratio, main memory usage, and compression speed for a variable number of references. This idea behind FRESCO’s second-order compression was recently extended in [19]. The authors report very high compression ratios, once the number of (compressed) reference sequences is increased.

There are other areas in Bioinformatics where compression is highly relevant, for instance, read compression [37–40] and compression of aligned sequences [41]. Very recently, several techniques for searching compressed representations of highly-similar sequences were proposed [42–46]. These tools provide efficient search capabilities based on an index much smaller than the raw sequence data. Note that most of these techniques [42, 44–46] are based on a multiple sequence alignment, and thus their real strength is the compressed representation of an index structure over all sequences. Our paper addresses the problem of finding a small compressed representation of all sequences, without the need of computing a multiple sequence alignment. In fact, indexes over collections of highly-similar sequences can benefit from our analysis of multi-reference compression algorithms, by building smaller indexes faster.

### 4.2 Summary

**Fig 9:**
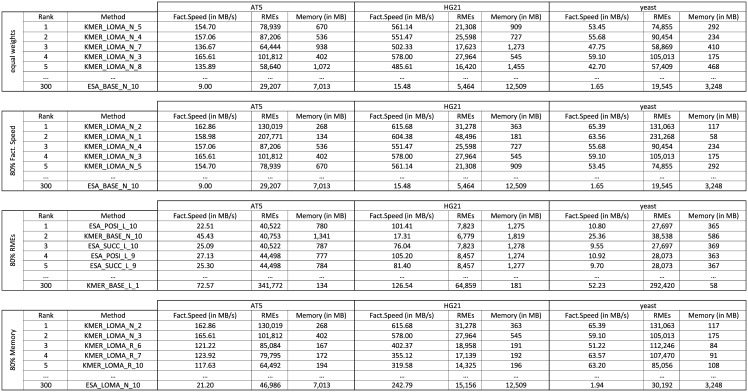
Ranking of the Top-5 and worst method for four scenarios: 1) equal weighting factors, 2) preference on factorization speed, 3) preference on small factorizations, and 4) preference on low memory footprint. We show the original criteria values for three datasets (AT5, HG21, yeast).

Our evaluation gives an overview over the wide range of performances for 30 factorization techniques against multiple references. If a user, however, has to select a method for factorization, it is not clear which one is actually the best method. The selection of a good factorization method is difficult and depends on multiple criteria: Expected factorization speed, optimality guarantees, and required main memory during factorization. Therefore, the selection of a factorization technique is a typical multi-criteria decision analysis (MCDA) problem. We solve the following MCDA-problem below: *Given up to 10 references and preferences weights on factorization size, factorization speed, and used main memory, which method is appropriate and how many references should be used?* We used a commonly used MCDA technique TOPSIS [47, 48] to find solutions to the following four scenarios: 1) overall best solution, 2) preference on small factorizations, 3) preference on fast factorization, and 4) preference on a small memory footprint. We analyzed 300 configurations, by associating the number of references with each of the standard configurations, for instance, CST_LOMA_N_2 is CST_LOMA_N with two references. For each of the 300 configurations we recorded the number of RMEs, factorization speed (in MB/s), and memory footprint (in MB) for each of the six datasets. The result is shown in Fig 9; for brevity we show the ranks and values for AT5, HG21, and yeast only.

The first scenario (equal weighting factors) attempts to select an overall good factorization method. The best ranked methods all use a (fast) KMER index and local matching for a medium number of references. The worst method is ESA_BASE_N_10, which uses a large ESA index, non-optimized matching and ten references without extension. When we increase the weight for factorization speed (from 33% to 80%), the KMER-based methods with local matching and only a few references are ranked best. If we set the priority on small factorizations, left-extension setups with optimizations (POSI/SUCC) are ranked best, with the exception of KMER_BASE_N_10, which yields a factorization close to the optimum, with medium memory usage and medium factorization speed. Finally, methods with KMER and local-matching are also ranked best for factorization with small main memory usage. If we further increase the preference weights to 99%, we obtain the following results: KMER_LOMA are the best methods for fast factorization, ESA_POSI and ESA_SUCC are the best methods for small factorizations, and CST_BASE methods are best for a low memory footprint.

## 5 Conclusions

We have devised and implemented referential sequence factorization against multiple references. Dissection of the framework into its components led to 30 configurations which were evaluated on six datasets from three species. Our analysis shows a wide range of factorization rates, factorization speed, and main memory usage. We identified the best configurations depending on common use cases.

One direction for future work is to a develop multi-reference factorization algorithm using recently developed multi-genome indexes [43, 44]. These indexes exploit similarities among sequences (here: the references) and provide different types of search functionality, as exact subsequence matching or approximate pattern matching. Generating such a multi-reference index over a set of references enables to compress sequences against these references. However, developing a fast and optimal compression algorithm for these indexes is a challenging problem. Another line of research is to investigate the combination of left-extension and right-extension techniques into one algorithm. The difficulty here is to decide which previously factorizations should be replaced whenever a new match in the factorized references is found, since there is not only one direction (left-right or right-left) during the factorization process. Furthermore, since the encoding of factors determines the compression ratio, it would be interesting to analyze and understand different encoding techniques on top of optimal factorizations.

## Appendix

### 5.1 Parameter analysis

**Fig 10:**
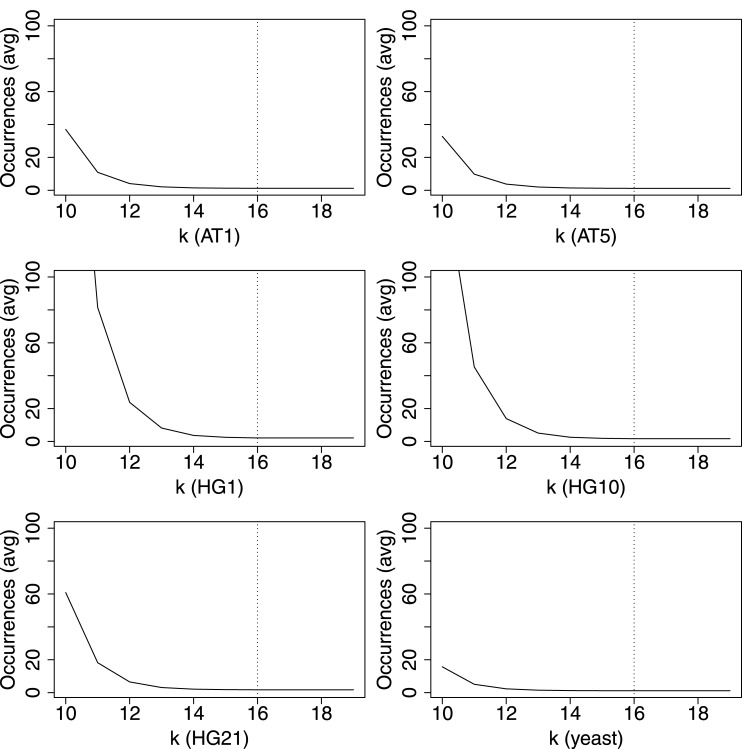
Average occurrences count of *k*-mer instances with varying *k*. For all six datasets independently, a value of *k* = 16 guaranteed that each 16-mer had an average number of occurrences smaller than 2 in the sequences.

We evaluated parameters for indexing and match finding techniques first. The choice of *k* for a *k*-mer index (*k*-mer indexes are used for referential compression as described in [18]) has a significant impact on factorization speed and factorization rate: too small *k* will lead to high verification costs, since a *k*-mer can occur very frequently in the references and too large *k* will increase the number of missed matches. In Fig 10, we show the average number of occurrences of all *k*-mers for our datasets depending on *k*. We have analyzed a range of 10 ≤ *k* ≤ 20. If we choose *k* ≥ 16, most *k*-mers are unique (note that the curve is growing exponentially to the left; see for instance HG1). We decided to set *k* = 16: then the average number of occurrences for *k*-mers is less than 2 for each dataset.

**Fig 11:**
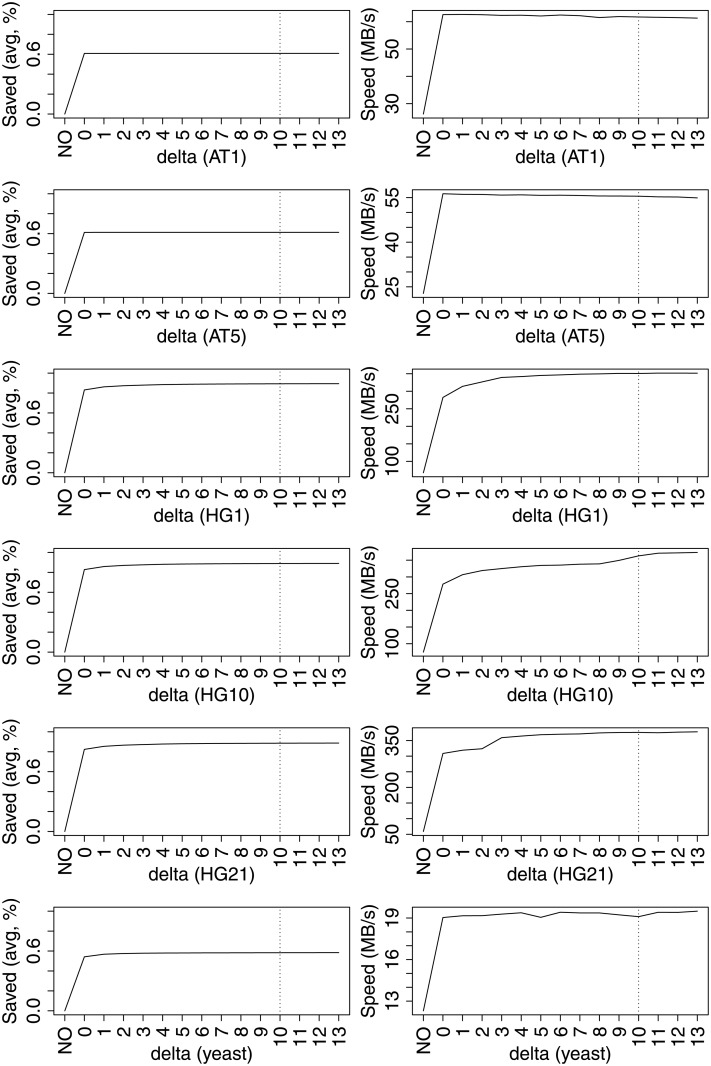
Percentage of index lookups saved against baseline BASE (left) and factorization speed (right). Larger *δ*-values reduce the number of index lookups and often increase factorization speed. A value of *δ* = 10 allows fast factorization for yeast and Human genomes, while not degrading factorization speed of AT too much.

Next we chose a *δ* value for our local match-finding algorithm. In general, a small *δ* will only identify SNPs (or very short indels), while a larger *δ* lead to increased verification times, since a larger neighborhood of the previous match end have to be compared to the to-be-factorized sequence prefix. The results of our analysis are shown in Fig 11. We compare the basic greedy match finding algorithm (denoted with NO) to a range of 0 ≤ *δ* ≤ 13. For all datasets, setting *δ* = 0, which only identifies SNPs, saves 50% or more of the index lookups. For Human chromosomes, more than 80% of the index lookups can be saved, since many sequence deviations in Human chromosomes from the 1000 Genome Projects are SNPs. The factorization speed is increased by a factor of almost 2 (yeast, AT) to factor of 3 and more (HG). For Human genomes, *δ* larger than 0 further avoids index lookups (and increase factorization speed), by detecting short indels. For the other datasets, factorization speed remains stable (yeast) or is slightly decreasing (AT). Our results for AT show that local match finding can reduce the factorization speed, if no short indels can be found and neighborhoods of previous matches are verified without success. We chose *δ* = 10 for our remaining experiments. For the algorithm in Table 3, we set *l*_*min*_ = *k* = 16, since this avoids spurious matches in the sequence.

**Fig 12:**
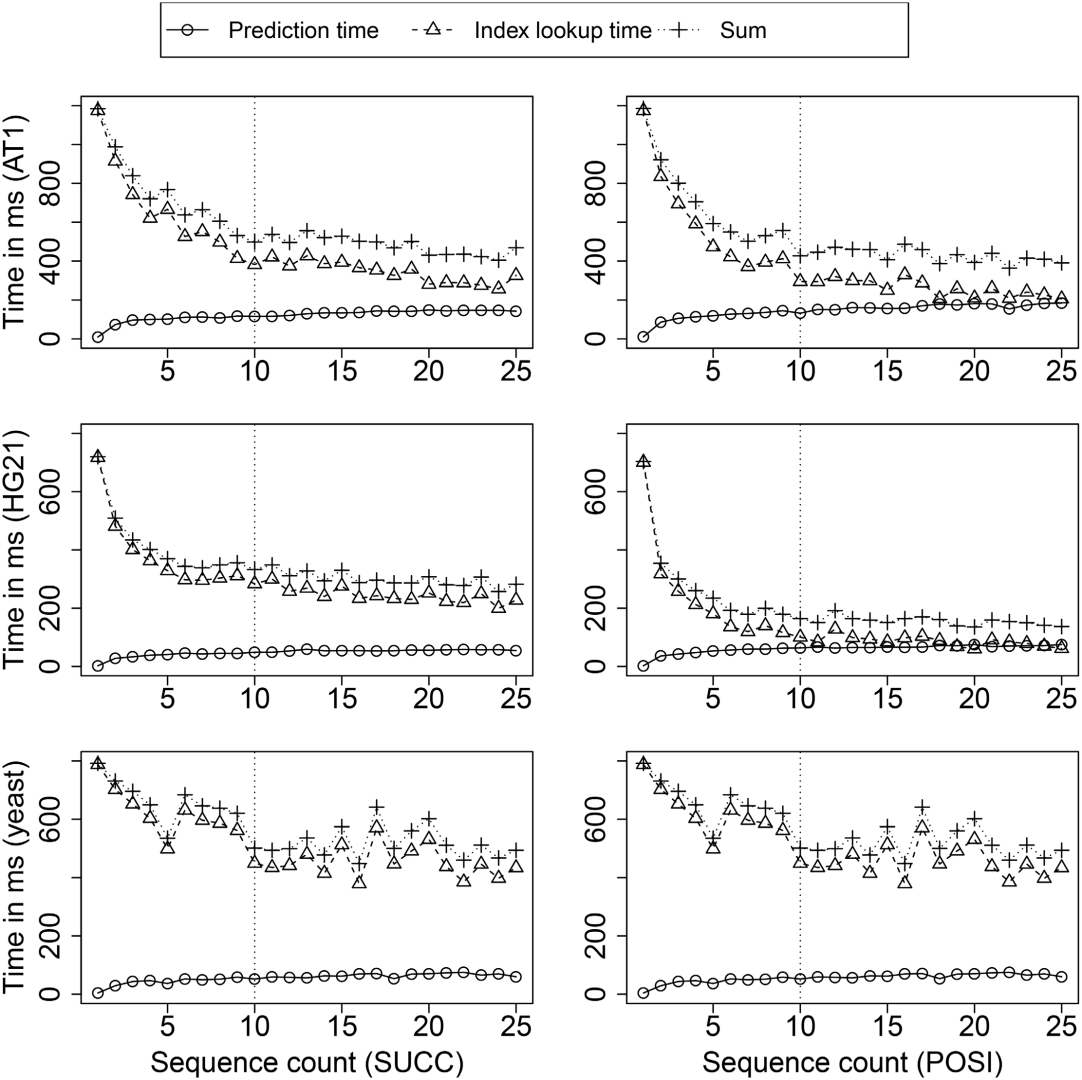
Effectiveness of SUCC/POSI on factorization time for an increasing number of sequences in the RME graph. A number of 10 sequences allows for fast factorization, while keeping the memory overhead low.

Next we analyzed the effect of RME graphs on factorization speed. Our analysis showed that it is not beneficial to store the RME graph of all previously factorized sequences for two reasons. First, the memory usage is linearly increasing with each sequence. Second, the time spent on checking RME graph predictions increases with the number of sequences in the RME graph. We measured the time spend on SUCC/POSI prediction and index lookups separately for an increasing number of factorized sequences, to find out how many sequences are actually necessary. The result is shown in Fig 12. For all datasets, RME prediction based on the RME graph shows a significant reduction of factorization time for the first few sequences. More than 10 sequences in the RME graph do not improve factorization times significantly. In fact, we found that the required number of index lookups is roughly constant with more than 10 sequences in the RME graph. Overall, POSI reduces the factorization time further for HG21 and all other human genomes (data not shown), but for AT and yeast, there is no significant speedup from SUCC to POSI. It seems that for these not-so-similar datasets, prediction based on the previous RME (SUCC) is already sufficient.
